# Brooklyn City Hospital

**Published:** 1860-08

**Authors:** James M. Minor

**Affiliations:** Attending Surgeon


### BROOKLYN CITY HOSPITAL.

*Cases Illustrating a new and Successful Treatment of Varicose veins, by the injection of Persulphate of Iron.*

BY JAMES M. MINOR, M. D., ATTENDING SURGEON.

The following cases possess the double interest of novelty and practical utility.

There are none of a similar character on record, except those in which this treatment was adopted subsequently to, and in imitation of them.

It will be observed that I have introduced a case of *aneurism*, treated with injections of the perchloride of iron, among cases of varicose veins, treated with the persulphate.

In doing so, I have violated the harmony of pathological relation, in order to illustrate the efficiency and innocuousness of the preparations of iron. The first case was in private practice, the others were treated in Hospital.

Case 1.—*Popliteal Aneurism cured by the Injection of the Perchloride of Iron.*—On the 9th day of November, 1857, I was requested by Dr. Jas. Crane to see Mrs. T. I found a small, pulsating, superficial, aneurismal sac, between the right labium and thigh, about the diameter of a Madeira nut, and projecting about half an inch above the surface. From it projected a small nipple-like, or rather tubular, offshoot, from which, previous to its ligation by Dr. Crane, arterial blood spouted *per saltum*. Mrs. T.'s account of it was, that about seventeen years previously she had received a severe blow at that point while entering a stage-coach, from the heavy iron hook attached to one end of the "back strap" of the middle seat, causing very severe pain at the time, but of short duration. Is not absolutely sure how long she has felt pulsation, but thinks that about a year since it became very distinct, and assumed the purplish tinge it now has; pulsation was more active at every menstrual period. A careful examination, by alternate pressure upon the femoral, and at a point posterior to the sac, shows a supply trunk, probably from some one of the perforating branches of the profunda femoris in front, and the obturator behind.

Upon consultation between Drs. Crane, Isaacs, and myself, and at Dr. Isaacs' suggestion it was determined to use injections of powerful styptics. This course was adopted in view of the manifold difficulties in the way of an effort to tie the supply trunks.

There were four several attempts made, at intervals of about a week, with solutions of lactate, muriated tincture, and perchloride of iron, using at the same time Signoroni's tourniquet to control the circulation through the femoral artery, and lessen the tendency to wash away the newly-formed clot. It was impossible to exert much force in controlling the current from the obturator artery, as the finger alone could be used.

The solution of the perchloride alone sufficed, with aid of the tourniquet, and the recumbent posture, to effectually coagulate the blood and block up the sac. The pain caused by the perchloride was very severe, and continued for twelve hours, and was followed by considerable inflammatory action. It was completely successful, and Mrs. T. recovered with entire obliteration of the sac. The tourniquet was kept on for some days, being loosened at intervals, to lessen the intolerable pain caused by the pressure. The filling by granulation of the cavity left where the coagulum came away (which it did by ulceration) occupied some weeks.

The notes of this case having been lost, will account for the omission of some points of interest. They have been drawn out from memory, and by the aid of the patient.

The following cases of varicose veins, treated by the injection of persulphate of iron, occurred in the Brooklyn City

Hospital, the notes of which are furnished me by R. P. Moore, M. D., House-Surgeon.

CASE 2.—*Varicose Veins of Leg—Injection of Persulphate of Iron—Cured.*

John Towle, admitted on March 1st, 1859, (Dr. Enos on duty), with ulcer from varicose vein on leg, of five years' duration; it has healed repeatedly, but again re-opened. Ordered poultice, and rest in recumbent posture.

April 25th.—Ulcers nearly healed. Injected liquor ferri persulphat. gtt. x.*

May 2d.—Veins obliterated at point of injection; neigboring branches still viracose.

May 20th. Ulcers entirely healed, and patient permitted to go out on a pass. Returned drunk, with abrasion of newly cicatrized surface.

June 13th.—Discharged cured.

CASE 3.—*Varicose Veins of Scrotum—Injection of persulphate of Iron—Cured.*

J. T., aged 22, American, admitted under Dr. Minor, Oct. 24th 1859, with varicose condition of scrotal veins of left side. Has enjoyed very good general health. For six months past has suffered much pain from distended veins of scrotum, extending through spermatic cord to inguinal canal of that side, and also in the testicle; can obtain no relief except in recumbent posture. Ordered cathartic. Suffers with languor and debility from involuntary seminal emissions, after which the pain is much aggravated.

Oct. 28th—Injected fourdrops of a solution of persulphate of iron (fourparts of water to one of persulphate) with Pravat's syringe, as modified by Tiemann. Patient was made to stand erect in order to fill the veins, and make them more distinct and prominent—a necessary precaution in such loose tissues as are found in that region. He fainted, but was soon restored by placing him in a recumbent posture. The operation scarcely caused any pain, either at the time or subsequently. A firm coagulum was formed in thirty seconds. Ordered cloths dipped in water to the part, and recumbent posture.

Nov. 3d—The clot formed by persulph. ferri gives indications of coming away by ulceration. Has felt less pain in cord since operation; nor does he feel any pain at the point of puncture.

---

* Officinal solution contains 43 per cent. of the solid persulphate.

Feb. 6th—Clot came away last night, leaving a healthy granulating surface.

26th—Discharged cured.

CASE 4.—*Varicose Veins of Scrotum—Second Injection—Cured.*

James Taylor was admitted a short time after his discharge in November last, with varicose condition of other deep scrotal veins near the cord. The veriform mass of enlarged veins around the point of former operation are entirely obliterated. Has been variously treated since second admission, but without resort to operative measures.

Feb. 14th—Veins increasing in size, attended with pain. Injected three drops of a solution of persulphate of iron in the proportion of one part persulphate to two of distilled water, followed by immediate coagulation of blood, as on former occasion, and with as little pain.

15th—Injection seems to have entirely relieved the pain in the cord, and he expresses himself as feeling better in every particular.

19th—Continues comfortable. Some pain and heat at the point of puncture, where there is an exceedingly hard and prominent tumor. Tumor is close to the cord, and seems in some measure to involve it. Seminal emissions occur at long intervals now. Cold water dressings.

26th—Clot decreasing in size, but still very hard. No appearance of ulcerating, as on former use of the persulphate.

March 1st—Tumor has steadily decreased in size; but little hardness remains. Veins completely obliterated when injected, as well as all others which were enlarged.

CASE 5.—*Varicose Veins of Leg—Injection of Persulphate of Iron—Cured.*

Carl de Buke, admitted Dec. 22d, 1859 with paronychia of left thumb. Varicose veins in left leg, which he has had for many years. Veins very much distended at one point. Owing to the size of the veins it was thought necessary to insert a larger quantity of the solution than usual.

Feb. 11th—Ten drops of a solution of the strength of one part persulphate to three of water, was used.

12th—A clot has formed, and obstructed the vein, though it does not appear to be so firm as in previous cases.

14th—Complains of pain at point of puncture, where there is a considerable swelling and redness. Apply cold lotion.

16th—Inflammation and pain subsiding. Continue lotion. No constitutional disturbance at any time.

22d—Tumor lessening in size, and redness disappearing.

March 10th—All inflammatory symptoms have subsided, and the vein is obliterated at point of operation.

Case 6.—*Varicose Veins of Leg—Injection of Persulphate of Iron—Cured.*

James Flemming was admitted Dec. 29th, 1859, with secondary syphilis, and ulcers on right leg; has varicose veins of the same leg, which are increasing in size, and he expresses a wish to be operated on for their relief.

Feb. 11—Injected as usual, three drops of a solution of the persulphate of iron, one part to four of water. A second puncture was made below the first.

13th—Coagulum formed, but not so marked as in other cases. No inflammation about punctures.

19th—Ulcer on leg has improved rapidly since operation.

25th—Vein obliterated between points of operation.

27th—Old ulcer cicatrized, and he desires to leave the hospital. Discharged cured.

It may be desirable to state briefly, the mode of procedure in the injection of varicose veins. A Pravat's syringe as modified by Mr. Tiemann, is the instrument used. This is a very small syringe of vulcanized rubber having a small (almost capillary) canula screwed to its lower end. This canula is cut obliquely at its extremity somewhat after the manner of a pen, ending in a sharp point. The piston rod is graduated to drops, to admit of the use of any quantity, however small.

The canula being screwed on, the quantity of the solution desired to be used is drawn in through the canula, which is then plunged into the vein, the patient standing erect. The finger of an assistant is then placed upon the vein, a little above and below the point of puncture, and firm pressure made; the piston is then forced down and the fluid injected. It is important that the pressure on the cardiac side of the puncture, should be sufficient to completely stop the upward current, as otherwise portions of the clot might be carried into the circulation. The pressure need be kept up for a minute or two only.

This completes the operation. The patient is placed in the recumbent posture, and cold water dressings applied, with directions not to rise for some days. The above mode of treatment of varicose veins, would seem to promise a safe, prompt, and painless cure, of a most uncomfortable, painful, and sometimes perilous complaint, for which, heretofore, there have been only uncertain and dangerous expedients.

The persulphate of iron, as far as heretofore used, seems to excite adhesive inflammation alone, thus avoiding that formidable affection, pyæmia; and I feel confident in recommending it to the profession, as a safe, simple, and almost certain remedy for varicose veins, and, with some qualifications, for *small* aneurisms.—*American Medical Times.*

☞ The visit of the Chicago Zouaves to our Eastern cities may properly be regarded with interest by physicians and sanitarians, as being happily adapted to awaken greater attention to the subject of physical education. Here is a company of young geutlemen, who, by strict adherence to physiological laws in all their personal habits, no less than in their special manual exercises and military evolutions, have acquired such dexterity, muscular strength, agility, and astonishing powers of physical endurance, that the press, the populace, and the military of New York, can scarcely find words to express the universal admiration and surprise which this young military corps has excited among us. How different this from the degrading engagements of acrobat showmen and the pugilists both in the results to the actors and the effects upon community! The time and means expended by these young men have amply been rewarded in the increase of enduring physical energies in their own persons, while their wonderful agility and their perfection of *physique*, as manifested in all their manœuvres, fascinate all beholders, and strongly confirm the opinion, that "the *zouave-iter in modo* is *fortiter in re*," no less for the individual man than in the active service of the military corps. Perfect ease of posture and complete command of every muscle are strikingly exhibited in every movement of these men—the well-trained muscles and healthy frame of each individual soldier happily illustrating the theory of the the effectiveness of the Zouave corps in battle—viz. complete *unity of purpose* with perfect *individuality of action.* We recommend to young men both the gymnastic training and the peculiar physical habits of these citizen soldiers, especially their rigid abstinence from all that can intoxicate.—*American Medical Times.*

Resection of the entire Radius.—Dr. Carnochan presented a patient from whom the *entire radius* had been removed for necrosis. The bone was exsected from joint to joint. The motion of the hand is almost perfect, the patient being able to write with facility and rapidity. The hand is not quite in its natural axis, as it bends a little inward, while the styloid process of the other side forms a prominent projection. Dr. Carnochan claims this as the first successful operation of this kind, and offers a premium of one hundred dollars to any one who can fairly disprove his claim. In conclusion he remarked that Dr. Gross of your city, was aware of this case, but he had chosen to omit all reference to it in his late work upon surgery.

The Death-bed of an Anatomist.—Retzius, the great Swedish anatomist, whose death was recently announced, while dying made observations on the progressing dissolution of his own body. His last words to his attendants were—"The struggle of death is hard, but it is of the highest interest to note this wrestle between life and death; now the legs are dead, now the muscles of the bowels cease their functions; the last struggle must be heavy, but for all that it is highly interesting."

Ages of Pregnant Women.—Dr. Granville, in some extensive statistics presented to the London Obstetrical Society, states that English women arrive at the culminating period of prolificacy at the age of thirty years, and French women at twenty-eight.

Maternal Influence on Fœtal Deformity.—At the last meeting of the London Obstetrical Society, Dr. Druitt, while discussing this subject, asked facetiously whether deformities of chickens could be accounted for by fright to the hen while sitting on the eggs.

It is calculated that the entire world of smokers, snuffers and chewers, consume 2,000,000 tons of tobacco annually, or 4,480,000,000 of pounds weight.

